# Performance Evaluation of Kits for Bisulfite-Conversion of DNA from Tissues, Cell Lines, FFPE Tissues, Aspirates, Lavages, Effusions, Plasma, Serum, and Urine

**DOI:** 10.1371/journal.pone.0093933

**Published:** 2014-04-03

**Authors:** Emily Eva Holmes, Maria Jung, Sebastian Meller, Annette Leisse, Verena Sailer, Julie Zech, Martina Mengdehl, Leif-Alexander Garbe, Barbara Uhl, Glen Kristiansen, Dimo Dietrich

**Affiliations:** 1 University Hospital Bonn (UKB), Institute of Pathology, Bonn, Germany; 2 Berlin Institute of Technology (TU Berlin), Biotechnology, Berlin, Germany; UT MD Anderson Cancer Center, United States of America

## Abstract

DNA methylation analyses usually require a preceding bisulfite conversion of the DNA. The choice of an appropriate kit for a specific application should be based on the specific performance requirements with regard to the respective sample material. In this study, the performance of nine kits was evaluated: EpiTect Fast FFPE Bisulfite Kit, EpiTect Bisulfite Kit, EpiTect Fast DNA Bisulfite Kit (Qiagen), EZ DNA Methylation-Gold Kit, EZ DNA Methylation-Direct Kit, EZ DNA Methylation-Lightning Kit (Zymo Research), innuCONVERT Bisulfite All-In-One Kit, innuCONVERT Bisulfite Basic Kit, innuCONVERT Bisulfite Body Fluids Kit (Analytik Jena). The kit performance was compared with regard to DNA yield, DNA degradation, DNA purity, conversion efficiency, stability and handling using qPCR, UV, clone sequencing, HPLC, and agarose gel electrophoresis. All kits yielded highly pure DNA suitable for PCR analyses without PCR inhibition. Significantly higher yields were obtained when using the EZ DNA Methylation-Gold Kit and the innuCONVERT Bisulfite kits. Conversion efficiency ranged from 98.7% (EpiTect Bisulfite Kit) to 99.9% (EZ DNA Methylation-Direct Kit). The inappropriate conversion of methylated cytosines to thymines varied between 0.9% (innuCONVERT Bisulfite kits) and 2.7% (EZ DNA Methylation-Direct Kit). Time-to-result ranged from 131 min (innuCONVERT kits) to 402 min (EpiTect Bisulfite Kit). Hands-on-time was between 66 min (EZ DNA Methylation-Lightning Kit) and 104 min (EpiTect Fast FFPE and Fast DNA Bisulfite kits). Highest yields from formalin-fixed and paraffin-embedded (FFPE) tissue sections without prior extraction were obtained using the innuCONVERT Bisulfite All-In-One Kit while the EZ DNA Methylation-Direct Kit yielded DNA with only low PCR-amplifiability. The innuCONVERT Bisulfite All-In-One Kit exhibited the highest versatility regarding different input sample materials (extracted DNA, tissue, FFPE tissue, cell lines, urine sediment, and cellular fractions of bronchial aspirates, pleural effusions, ascites). The innuCONVERT Bisulfite Body Fluids Kit allowed for the analysis of 3 ml plasma, serum, ascites, pleural effusions and urine.

## Introduction

DNA methylation of cytosines within the CpG dinucleotide context is an epigenetic mechanism, which plays an important role in biological processes, such as cell differentiation and development [1]. Furthermore, aberrant DNA methylation is a hallmark of malignant tumors and plays a key role during carcinogenesis [2]. Studies on DNA methylation changes in the course of cancer development and progression will broaden the understanding of this devastating disease and will lead to several clinically relevant biomarkers and therapy approaches in the future. A few DNA methylation biomarkers are already on the road to clinical use for predictive, diagnostic and screening purposes. Methylation of the promoter of the *MGMT* gene in gliomas allows for the prediction of the response to alkylating agents [3]. The *MGMT* promoter methylation status has become a parameter for stratification of patients with glioma within several clinical trials [4]. Macrodissected tumor tissues from sections of FFPE tumors are the sample of choice to achieve good results [4]. Two additional tests based on the methylation analysis in FFPE tissues already show a high level of validation qualifying them for clinical use. The ConfirmMDx test (MDxHealth, Inc., Irvine, CA, USA) is based on DNA methylation of *RASSF1*, *APC*, and *GSTP1* in FFPE biopsies [5] and intends to help distinguish patients who have a true negative biopsy from patients who may have occult prostate cancer. DNA methylation of *PITX2* in FFPE prostatectomy specimens is a strong prognostic biomarker for identifying patients who are at high risk to suffer from prostate-specific antigen (PSA) recurrence after radical ectomy [6], [7], [8], [9]. Free-circulating methylated *SEPT9* gene copies in plasma as a screening biomarker for colorectal cancer were recently validated in a large observational prospective screening trial including more than 7,000 asymptomatic subjects [10]. *SHOX2* DNA methylation is another plasma based biomarker that allows for the identification of lung cancer [11]. Furthermore, *SHOX2* DNA methylation is a validated biomarker for detecting lung cancer in the cellular fraction of bronchial aspirates [12], [13] and pleural effusions [14], [15] as well as in EBUS-TBNA (endobronchial ultrasound with transbronchial needle aspiration) specimens [16]. These examples of methylation biomarkers with the highest level of validation clearly indicate the necessity of technologies, which allow for the accurate determination of DNA methylation in various sample types. These sample types each represent their specific technological challenges, i.e. DNA fragmentation in FFPE tissues and low abundance of methylated copies in blood plasma. The availability of kits and tools to measure DNA methylation in these sample types is mandatory to open this research area to a wide group of researchers.

Methylated cytosine exhibits a similar base pairing behaviour as cytosine and therefore methlyated and unmethylated cytosines are difficult to distinguish from each other by conventional hybridization-based molecular biological methods, i.e. microarrays and PCR. In 1992, Frommer *et al.* [17] published a protocol, which allowed for a positive display of 5-methylcytosine. In this protocol DNA was denatured using NaOH and subsequently incubated for 16 hours in the presence of 3.1 M sodium bisulfite and 0.5 M hydroquinone in order to achieve a deamination of cytosines to uracils while methylated cytosines remain unaffected. Consequently, the epigenetic information of the DNA is transformed into sequence information, which can be studied via PCR and other hybridization based methods. Today, direct detection of DNA methylation, without bisulfite conversion, through single-molecule, single molecule real-time (SMRT) sequencing [18] is possible. Nevertheless, the specific conversion of cytosines to uracils by means of bisulfite is still state of the art in DNA methylation analyses. However, several technological advances have now led to protocols which are much more convenient and user friendly compared to the original 16 hours protocol [19], [20], [21], [22], [23], [24]. In the meantime, numerous kits are commercially available allowing methylation analyses even for inexperienced researchers. Kits are available from Qiagen GmbH (Hilden, Germany), Zymo Research (Irvine, CA, USA), Millipore (Billerica, MA, USA), Analytik Jena (Jena, Germany), New England Biolabs (Ipswich, MA, USA), Promega (Fitchburg, WI, USA), Thermo Scientific (Waltham, MA, USA). All kits are designed to enable the efficient bisulfite conversion of extracted high molecular DNA. However, only a few kits allow for the modification of DNA from challenging input sample material, i.e. fixed tissues and body fluids. Fixed tissues usually contain only degraded DNA which further suffers from crosslinking. Fixed tissues require an efficient cell lysis in order to release DNA of sufficient quality and quantity for downstream analyses. Body fluids often only contain low concentrations of DNA which needs to be concentrated prior to bisulfite conversion in order to reduce the volume therefore enabling the input into the bisulfite reaction. Only one product (innuCONVERT Bisulfite Body Fluids Kit, Analytik Jena) is suited to process large sample volumes of urine, ascites, pleural effusions, blood plasma and blood serum. The aim of this study is to evaluate the performance of the most widely used kits: EpiTect Fast FFPE Bisulfite Kit, EpiTect Bisulfite Kit, EpiTect Fast DNA Bisulfite Kit (Qiagen GmbH), EZ DNA Methylation-Gold Kit, EZ DNA Methylation-Direct Kit, and EZ DNA Methylation-Lightning Kit (Zymo Research). Furthermore, the innuCONVERT Bisulfite Body Fluids Kit (Analytik Jena) and related kits (innuCONVERT Bisulfite Basic Kit and innuCONVERT Bisulfite All-In-One Kit) were analyzed. **Table 1** provides an overview of the analyzed kits and their applicability to different sample types.

**Table 1:** List of the different kits for the preparation of bisulfite-converted DNA from various sources.

Kit	Sample Input Material	Sample Specification	Comment
EpiTect Fast DNA Bisulfite Kit (Qiagen GmbH)	Extracted DNA from fresh and fixed tissues.	max. 20 μl extracted DNA; 1 ng –2 μg DNA	EpiTect Fast DNA Bisulfite Kit and EpiTect Fast FFPE Bisulfite Kit share the same core kit.
EpiTect Fast FFPE Bisulfite Kit (Qiagen GmbH)	Extracted DNA from fresh and fixed tissues. Direct input of FFPE tissue sections.	max. 20 μl extracted DNA; 1 ng –2 μg DNA; 1×10 μm FFPE tissue sections	EpiTect Fast DNA Bisulfite Kit and EpiTect Fast FFPE Bisulfite Kit share the same core kit.
EpiTect Bisulfite Kit (Qiagen GmbH)	Extracted DNA from fresh and fixed tissues.	max. 20 μl extracted DNA; 1 ng –2 μg DNA	
innuCONVERT Bisulfite Basic Kit (Analytik Jena AG)	Extracted DNA from fresh and fixed tissues.	max. 50 μl extracted DNA; 500 pg –10 μg DNA	All three innuCONVERT Bisulfite kits share the same core kit.
innuCONVERT Bisulfite All-In-One Kit (Analytik Jena AG)	Extracted DNA from fresh and fixed tissues. Direct input of FFPE tissue, cell lines, fresh tissues and aspirates.	max. 50 μl extracted DNA; 500 pg –10 μg DNA; max. 3×10 μm FFPE tissue sections; max. 10 mg FFPE tissue; max. 1 mg fresh or frozen tissue; max. 5×10^5^ cultured cells; Cellular fractions of bronchial aspirates, pleural effusions, ascites; Urine sediment	All three innuCONVERT Bisulfite kits share the same core kit.
innuCONVERT Bisulfite Body Fluids Kit (Analytik Jena AG)	Extracted DNA from fresh and fixed tissues. Direct input of blood plasma and serum.	max. 50 μl extracted DNA; max. 3 ml blood plasma, serum, ascites, pleural effusions or urine	All three innuCONVERT Bisulfite kits share the same core kit.
EZ DNA Methylation-Gold Kit (Zymo Research, Inc.)	Extracted DNA from fresh and fixed tissues.	max. 20 μl extracted DNA; 500 pg –2 μg DNA	
EZ DNA Methylation-Direct Kit (Zymo Research, Inc.)	Extracted DNA from fresh and fixed tissues. Direct input of FFPE tissue sections, cultured cell lines, fresh tissues and whole blood.	max. 20 μl extracted DNA; 50 pg –2 μg DNA; FFPE tissue sections; max. 0.1 mg fresh or frozen tissue; 10–10^5^ cells; max. 0.5 μl whole blood	
EZ DNA Methylation-Lightning Kit (Zymo Research, Inc.)	Extracted DNA from fresh and fixed tissues.	max. 20 μl extracted DNA; 500 pg –2 μg DNA	

The kit performance was compared with regard to conversion efficiency, DNA yield, DNA degradation, purity, stability and handling. The suitability for different starting materials, i.e. extracted DNA from fresh tissues as compared to DNA from FFPE tissues, sections from FFPE tissues without prior extraction, tissue lysates, cell line pellets and body fluids, such as urine, ascites and pleural effusions, were tested. The performance was tested by means of real-time PCR (yield, purity, stability), clone sequencing (conversion), UV (purity and yield), HPLC (inappropriate conversion), and gel electrophoresis (DNA degradation).

## Materials and Methods

### Ethics Statement

The study has been approved by the Institutional Review Board (IRB) at the University Hospital of Bonn. Informed consent (written) was obtained from all donors.

### Sample and DNA Preparation

All clinical specimens and samples were obtained from the University Hospital of Bonn, Germany.

For the preparation of plasma and serum blood samples were collected using 9 ml S-Monovette with EDTA K2 and Z Gel (Sarstedt AG & Co, Nuembrecht, Germany), respectively. Plasma and serum was prepared by two consecutive centrifugation steps (1350×g, 12 min and 3000×g, 12 min). Pleural effusion, ascites and urine samples were prepared by centrifugation at 4000×g for 10 min. Urine, ascites, pleural effusions, plasma and serum from 5 to 23 cancer patients were pooled.

The DNA was extracted from FFPE placental tissue and fresh placental tissue by means of proteinase K lysis and subsequent phenol-chloroform extraction. Five sections (10 μm each) from FFPE tissue specimens and 30 mg fresh placental tissue, respectively, were shredded. The samples were lysed overnight at 60°C with 450 μl lysis buffer (50 mM Tris pH 8.4, 1 mM EDTA, 0.5% [v/v] Tween20) and 50 μl proteinase K (2% in lysis buffer) at 1000 rpm in a thermomixer. After 6 h another 50 μl of proteinase K solution was added. The proteinase K was inactivated (30 min, 95°C) and the RNA was digested with 200 U RNase I (Ambion, Invitrogen, Carlsbad, CA, USA) for 30 min at 37°C. The DNA was extracted by phenol-chloroform extraction as previously described [25].

DNA from washed human research sperm (NW Andrology & Cryobank Inc., Spokane, WA, USA) and CpGenome Universal Methylated DNA were used as unmethylated and methylated reference DNA, respectively.

### Bisulfite Conversion

Bisulfite conversion and subsequent purification was performed according to the respective kit protocols. The following kits were applied: EpiTect Bisulfite Kit (Catalogue No. 59104), EpiTect Fast DNA Bisulfite Kit (Catalogue No. 59824), EpiTect Fast FFPE Bisulfite Kit (Catalogue No. 59844, Qiagen GmbH, Hilden, Germany), EZ DNA Methylation-Direct Kit (Catalogue No. D5020), EZ DNA Methylation-Gold Kit (Catalogue No. D5005), EZ DNA Methylation-Lightning Kit (Catalogue No. D5030, Zymo Research, Inc., Irvine, CA, USA), innuCONVERT Bisulfite Basic Kit (Catalogue No. 845-IC-1000040), innuCONVERT Bisulfite All-In-One Kit (Catalogue No. 845-IC-2000040), innuCONVERT Bisulfite Body Fluids Kit (Catalogue No. 845-IC-3000040, Analytik Jena AG, Jena, Germany).

For the analysis of inappropriate conversion 1 μmol T_5_^Me^CT_5_ oligonucleotides were applied to each bisulfite reaction. The bisulfite reactions were stopped with 50 μl NaOH (5 M) without subsequent purification.

### UV-Measurement

UV spectrophotometry was carried out using a Nanodrop ND-1000 spectral photometer (Nanodrop Technologies, Wilmington, DE, USA). For calculation of the DNA concentration the multiplication factor 33 was used for single stranded DNA (bisulfite DNA) and 50 for double stranded DNA (genomic DNA).

### qPCR (*SHOX2*/*SEPT9*/*ACTB* Triplex Assay and CFF Assay)

A methylation-specific triplex qPCR assay to determine *SHOX2* and *SEPT9* DNA methylation (using *ACTB* as reference) was performed as previously described [15].

A qPCR targeting a cytosine free fragment (CFF, GRCh37:Chr13,19555120–19555208) as previously described [26] was used to quantify the total amount of unconverted and converted DNA simultaneously (forward primer: taagagtaataatggatggatgatg, reverse primer: cctcccatctcccttcc, detection probe: 6-FAM-atggatgaagaaagaaaggatgagt-BHQ1). The total DNA yield compared to a calibrator sample (10 ng genomic DNA) was determined using the deltaCT method. PCR was performed using an AB 7500 Fast Real-Time PCR System (Life Technologies Corporation, Carlsbad, CA, USA) using a PCR buffer as previously described [15] and the following temperature profile: 20 min at 95°C followed by 50 cycles with 2 s at 62°C, 45 s at 56°C and 15 s, 95°C.

### HPLC Analysis

The oligonucleotides T_5_^Me^CT_5_ and T_11_ were quantified using HPLC-UV (Detection at 260 nm) using a DNAPac PA-100 (4×250 mm, Dionex, Sunnyvale, CA, USA) column applying an isocratic gradient of 62% buffer A and 38% buffer B (buffer A: 0.01 M NaOH, 1 M NaCl; buffer B: 0.01 M NaOH, 0.2 M NaCl) at a flow rate of 1.5 ml/min and 30°C.

### CFP Clone Sequencing Conversion Assay

The conversion efficiency was tested by means of a PCR product generated using primers targeting cytosine-free priming (CFP) sites. Two different PCR products (228 bp, GRCh37:Chr2,21454728–21454955 and 415 bp; GRCh37:Chr2,21557568–21557982) were amplified (228 bp-fragment forward primer: tgggttaaagtgattgagtaa, 228 bp-fragment reverse primer: tattcatccttcaacttaccct, 415 bp-fragment forward primer: atgggtaaggatatgaagttaat, 415 bp-fragment reverse primer: tatcacttaatcacctcctaaacta), cloned into the plasmid pCR2.1 using the TA Cloning Kit (Invitrogen, Carlsbad, CA, USA), and sequenced by Sanger Sequencing.

### Data Evaluation and Statistical Analysis

Statistical analyses were performed with SPSS software version 21 (IBM, Armonk, NY, USA). Performance differences between the compared bisulfite kits were examined by t-test and ANOVA. Correlation between conversion efficiency and inappropriate conversion was tested using Pearson correlation. P-values <0.05 were considered to be statistically significant. Two-sided p-values are reported.

## Results

### DNA Yield

**Figure 1:**
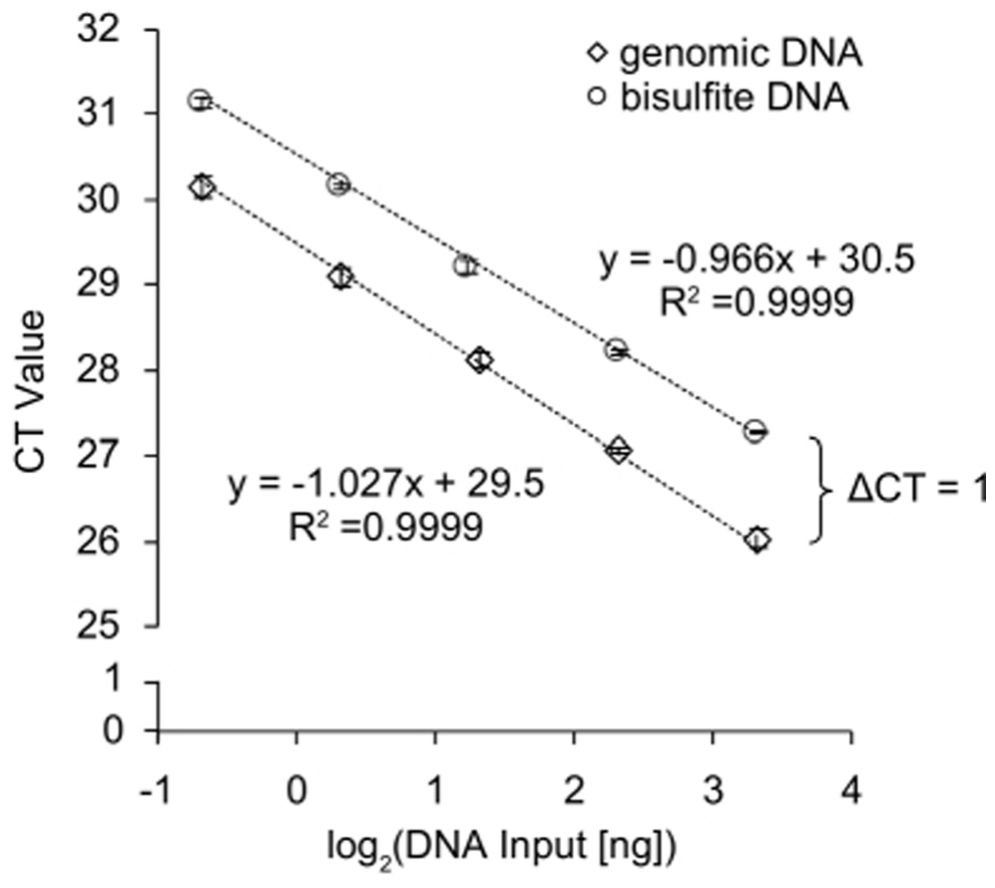
Analytical Performance of the CFF qPCR Assay. Quantitative real-time PCR analysis of a dilution series of genomic (unconverted) and bisulfite-converted DNA (10, 5, 2.5, 1.25, 0.625 ng per PCR reaction) using the CFF assay. The CFF amplicon is free of cytosines within the sense strand and therefore allows for the amplification of bisulfite-converted and genomic DNA. Shown are mean values (± standard deviation) of triplicate measurements. The assay showed a PCR efficiency of 2.0 for both templates.

**Figure 2:**
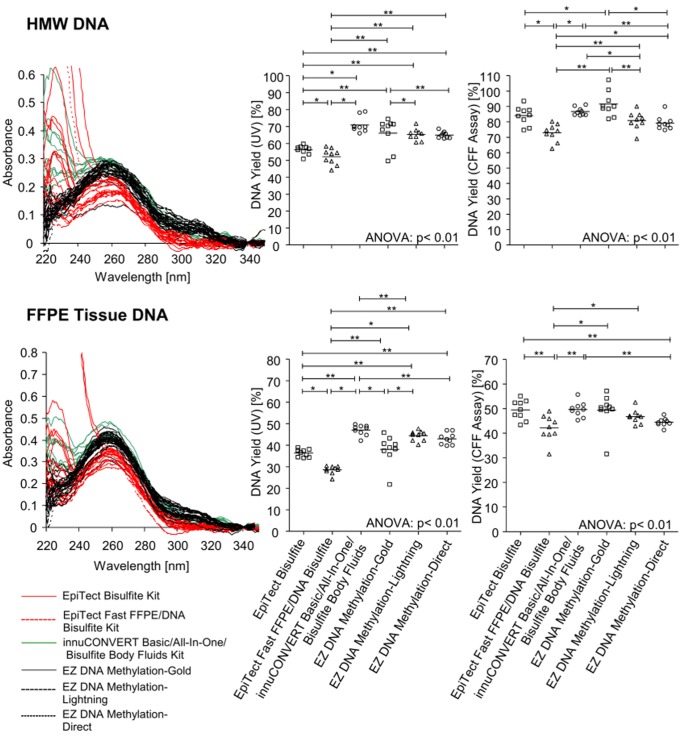
DNA Yield and Purity after Bisulfite Conversion Applying Nine Different Kits. HMW (**upper panel**) and FFPE tissue (**lower panel**) DNA was bisulfite-converted using nine different commercially available kits. Each bisulfite conversion was conducted in nine replicates for each kit. **Left panel:** UV spectra of bisulfite-converted and purified DNA (**left panel**). **Middle and right panel:** DNA yield of bisulfite-converted DNA as determined via UV (**middle panel**) measurement and via CFF qPCR assay (**right panel**), respectively. Shown are mean values of triplicate PCR measurements.

The DNA yield was determined by UV spectrophotometry and a quantitative real-time PCR assay measuring a cytosine free fragment (CFF). The CFF assay targets a locus containing no cytosines within the sense strand. Therefore, the sense strand of this locus is not altered during bisulfite conversion and can be used to quantify bisulfite as well as genomic DNA without the introduction of a bias. Accordingly, this assay allows for the direct comparison of genomic input DNA and bisulfite output DNA. The results obtained from bisulfite converted DNA were corrected by the factor 2 since in genomic DNA the sense and antisense of the double stranded DNA act as template whereas in bisulfite DNA only the sense strand can be amplified by PCR leading to a shift of 1 CT. The results show a robust assay suitable for accurate DNA quantification (**Figure 1**). Each kit was tested with high molecular weight (HMW) DNA extracted from fresh placental tissue and DNA extracted from FFPE placental tissue. The results are illustrated in **Figure 2** and summarized in **Table 2**. In summary, all kits showed high yields of bisulfite converted DNA ranging between 29% and 92%. DNA quantification using UV showed the lowest yield when applying the EpiTect Fast FFPE and EpiTect Fast DNA Bisulfite kits. Highest DNA yields both with HMW and FFPE tissue DNA were obtained using the innuCONVERT Bisulfite kit family. The DNA yield analyzed by real time PCR showed similar results. Accordingly, these kits are of particular usability when samples are processed which are expected to contain only minute DNA amounts, i.e. microdissected cells. Again the EpiTect Fast FFPE and EpiTect Fast DNA Bisulfite kits showed the lowest DNA yield both on HMW DNA and FFPE tissue DNA. With regard to HMW DNA the EZ DNA Methylation-Gold Kit yielded the highest DNA amounts while the innuCONVERT Bisulfite kit family showed the highest yield when applying FFPE tissue DNA.

**Table 2:** Overview over the kit performance results.

Kits	Yield from HMW Input DNA (UV) [%]	Yield from HMW Input DNA (CFF qPCR) [%]	Yield from FFPE Tissue Input DNA (UV)* [%]	Yield from FFPE Tissue Input (CFF qPCR)* [%]	Purity	Conversion Efficiency [%]	Inappropriate Conversion [%]	Integrity (Fragmentation)	Suitability for FFPE Tissue Sections	Suitability for High Volumes (Body Fluids)	Suitability for Tissue and Cells	Time-to-Result [min]*	Hands-on-Time [min]*	Storage Stability*
EpiTect Bisulfite Kit	56	84	36	49	+	98.7	1.4	+	NA	NA	NA	402	97	+
EpiTect Fast DNA Bisulfite Kit^‡^	52	73	29	42	++	99.8	2.0	++	NA	NA	NA	139	104	+
EpiTect Fast FFPE Bisulfite Kit^‡^	52	73	29	42	++	99.8	2.0	++	+	NA	++	139	104	+
innuCONVERT Bisulfite Basic Kit^†^	71	87	47	50	+	99.0	0.9	++	NA	NA	NA	131	86	++
innuCONVERT Bisulfite All-In-One Kit^†^	71	87	47	50	+	99.0	0.9	++	++	NA	++	131	86	++
innuCONVERT Bisulfite Body Fluids Kit^†^	71	87	47	50	+	99.0	0.9	++	NA	++	NA	131	86	++
EZ DNA Methylation-Gold Kit	66	92	38	49	++	99.7	2.5	−	NA	NA	NA	240	77	+
EZ DNA Methylation-Lightning Kit	65	81	44	47	++	99.8	2.0	++	NA	NA	NA	137	66	+
EZ DNA Methylation-Direct Kit	65	79	43	44	++	99.9	2.7	+	−	NA	+	297	76	+

*Starting material: extracted DNA.

†‡ These kits share the identical bisulfite conversion core kit and subsequent purification protocol.

**Figure 3:**
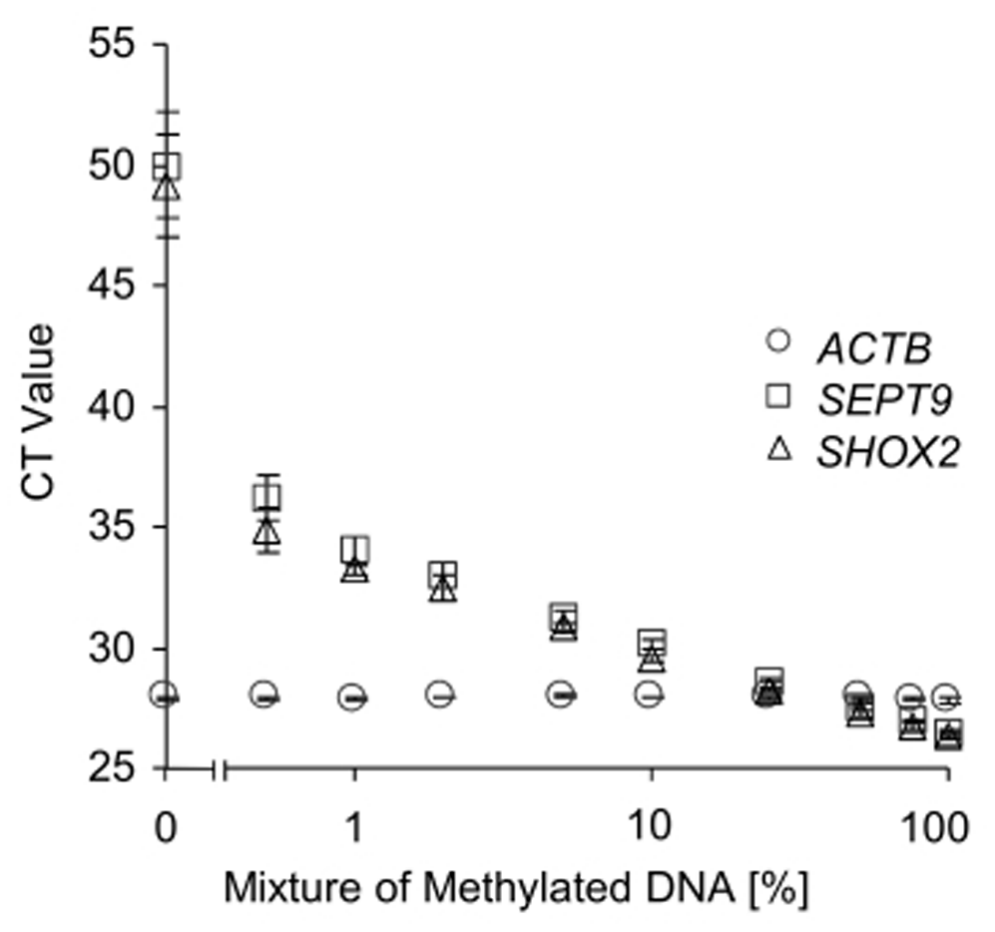
Analytical Performance of the *ACTB*/*SHOX2*/*SEPT9* Triplex Assay. Bisulfite-specific and methylation-specific quantitative real-time PCR analysis of mixtures of bisulfite-converted artificially methylated DNA and unmethylated DNA from sperm. The *ACTB*/*SHOX2*/*SEPT9* assay amplifies bisulfite-converted methylated *SHOX2* and *SEPT9* gene copies and total bisulfite-converted DNA using an *ACTB* amplicon. Shown are mean values (± standard deviation) of triplicate measurements.

Furthermore, a quantitative and sensitive methylation-specific triplex real-time PCR assay for determination of *SHOX2* and *SEPT9* DNA methylation was used to evaluate the kit performance. The determined *SHOX2* and *SEPT9* DNA methylation was referred to total DNA quantified using an *ACTB* assay. The analytical performance of the assay was characterized by measuring mixtures of methylated and unmethylated DNA (**Figure 3**). The *ACTB* assays showed the same CT value throughout the series of DNA mixtures as the applied *ACTB* primers are methylation-unspecific. In contrast, the *SHOX2* and *SEPT9* primers are methylation-specific and therefore amplify only methylated DNA. Accordingly, the CT values correlate with the relative amount of methylated DNA in the mixtures (**Figure 3**). Each kit was tested with high molecular weight (HMW) DNA extracted from fresh placental tissue and DNA extracted from FFPE placental tissue. Lowest yields of bisulfite converted DNA were obtained using the EpiTect Fast FFPE and EpiTect Fast DNA Bisulfite kits. Highest yield of bisulfite converted HMW DNA was obtained using the EZ DNA Methylation-Gold kit. The innuCONVERT kit family showed the highest yield when applying DNA from FFPE tissue (**Figures 2** **and** **4** **,** **Table 2**).

**Figure 4:**
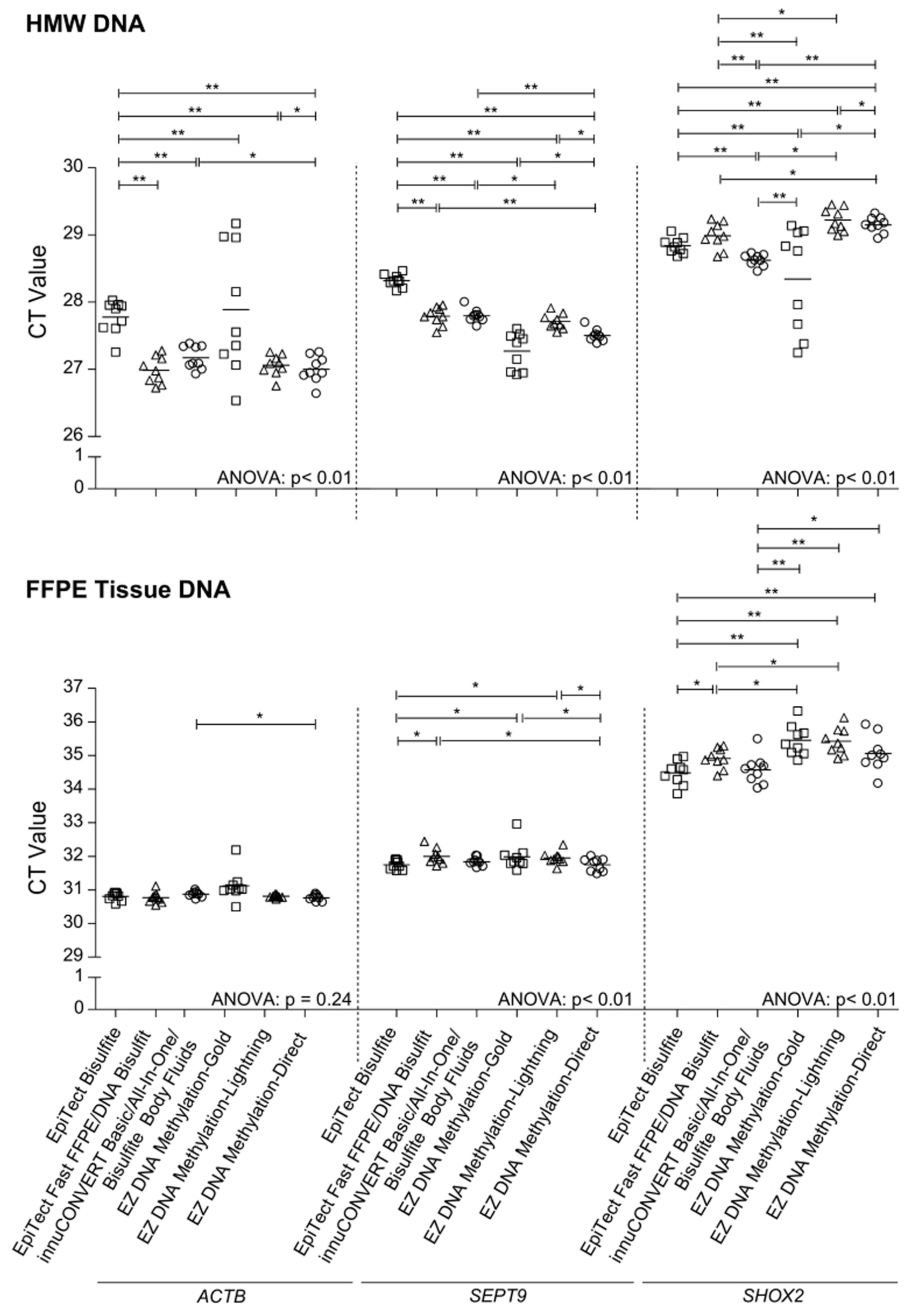
Kit Performance Comparison Using a Triplex qPCR (*ACTB*/*SHOX2*/*SEPT9*) Assay. HMW (**upper panel**) and FFPE tissue (**lower panel**) DNA was bisulfite-converted using nine different commercially available kits. Each bisulfite-converted DNA was assayed using the *ACTB*/*SHOX2*/*SEPT9* triplex qPCR asay. Shown are mean (± standard deviation) CT values of triplicate measurements. **Left panel:** *ACTB* CTs; **middle panel:** *SEPT9* CTs, **right panel:** *SHOX2* CTs.

### Integrity, Stability and Purity

**Figure 5:**
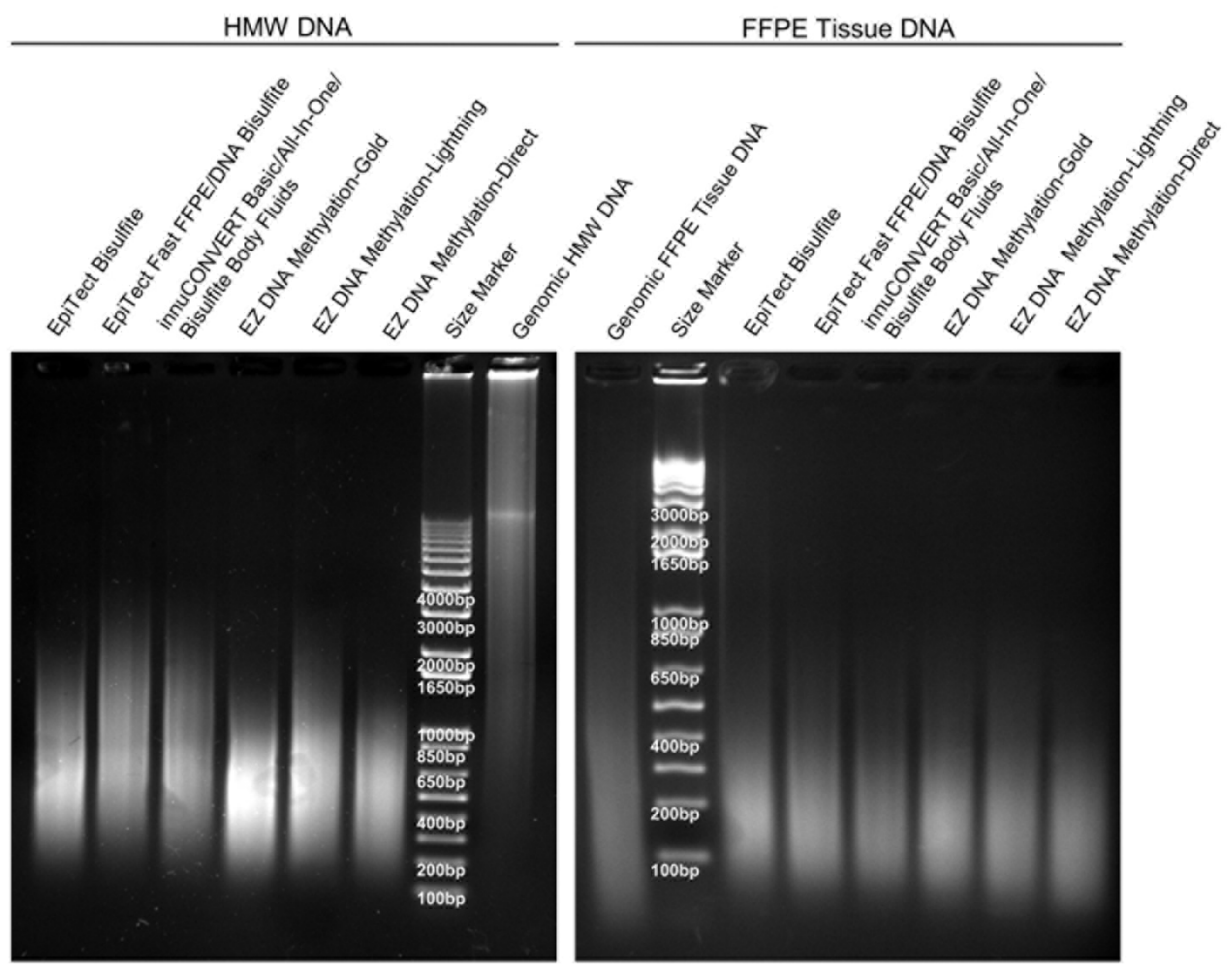
DNA Integrity after Bisulfite Conversion. Agarose gel electrophoresis of genomic and bisulfite-converted DNA (2 μg each). The bisulfite conversion was carried out using nine different kits. Shown is bisulfite-converted DNA from HMW (left) and FFPE tissue (right) input DNA. Each bisulfite-converted DNA represents a DNA pool from nine independent bisulfite reactions per kit.

The integrity of the bisulfite-converted HMW DNA as determined by means of gel electrophoresis revealed a visible difference between DNA samples prepared with the different kits (**Figure 5** **,** **Table 2**). All DNA samples showed degradation (fragmentation) compared to the genomic input DNA. The differences in fragmentation were particularly obvious in the case of the HMW DNA samples. The kits which are based on the longest duration of the bisulfite reaction (EZ DNA Methylation-Gold Kit, EZ DNA Methylation-Direct Kit, and EpiTect Bisulfite Kit) showed higher fragmentation compared to the kits which are based on a fast bisulfite conversion protocol (i.e. EpiTect Fast FFPE Kit, EpiTect Fast DNA Bisulfite Kit, and the InnuCONVERT Bisulfite kit family). The DNA from FFPE tissue already showed a strong fragmentation before bisulfite conversion, therefore only minor differences between the kits can be observed after conversion.

**Figure 6:**
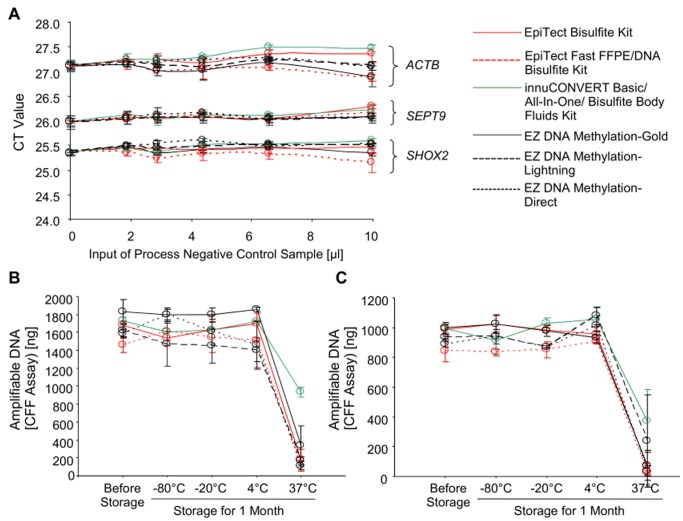
PCR Inhibition and Storage Stability. **A:** Inhibitory effect of eluate derived from different bisulfite conversion kits. Water was applied to nine different bisulfite conversion kits and processed like sample DNAs (process negative control sample). Different volumes (0–10μl) of this eluate were spiked into the *ACTB*/*SEPT9*/*SHOX2* q PCR (20 μl total PCR volume). Each PCR contained 10 ng bisulfite-converted methylated template DNA. **B, C:** Storage stability. Bisulfite-converted DNA was prepared from HMW (**B**) and FFPE tissue (**C**) input DNA using nine different bisulfite conversion kits. The bisulfite-converted DNA was stored for one month at −80°C, −20°C, 4°C, and 37°C, respectively. Total amount of intact (PCR-amplifiable) DNA was determined using the CFF qPCR assay. Each kit was tested in nine replicates. Each PCR was run in duplicate. Shown are mean values (± standard deviation).

The potential inhibitory effect caused by carry-over of impurities from the bisulfite reaction was tested in a spiking experiment. Water containing no DNA was processed through the bisulfite protocols of the different kits. These eluates (process negative control samples) were expected to contain similar impurities as DNA samples processed with the respective kits. Accordingly, these eluates could be used to quantify the inhibitory effect of impurities derived from the different kit protocols. Increasing volumes of these process negative controls were spiked into PCR reactions each containing a constant amount of template DNA. Accordingly, identical CT values are expected in all samples unless PCR inhibition leads to an increase of CT values. As shown in **Figure 6** (**A**) none of the bisulfite kits led to a significant inhibition of the PCR even at input volumes of 10 μl of process negative control sample into a 20 μl PCR reaction.

Bisulfite-converted DNA is mainly single-stranded DNA generated under harsh chemical reaction conditions which cause significant degradation. This DNA might further suffer from degradation due to storage. A high stability of bisulfite-converted DNA is required if studies are to be conducted over a period of time. To investigate the DNA stability a storage experiment was performed. Bisulfite-converted DNA samples prepared with the different kits were stored for 1 month at −80°C to 37°C. The amount of remaining intact DNA was determined using the CFF qPCR assay. Storage stability of bisulfite DNA derived from HMW and FFPE tissue DNA was tested (**Figure 6** **B, C**). The stability of DNA prepared with all kits was high when stored at −80°C, −20°C and 4°C compared to freshly prepared DNA. By means of qPCR no degradation of DNA was detectable. When stored at 37°C the amount of successfully PCR-amplified DNA decreased dramatically. The innuCONVERT Bisulfite kit family showed a significantly higher DNA stability at 37°C after 1 month compared to the other kits.

### Conversion Efficiency and Specificity

**Figure 7:**
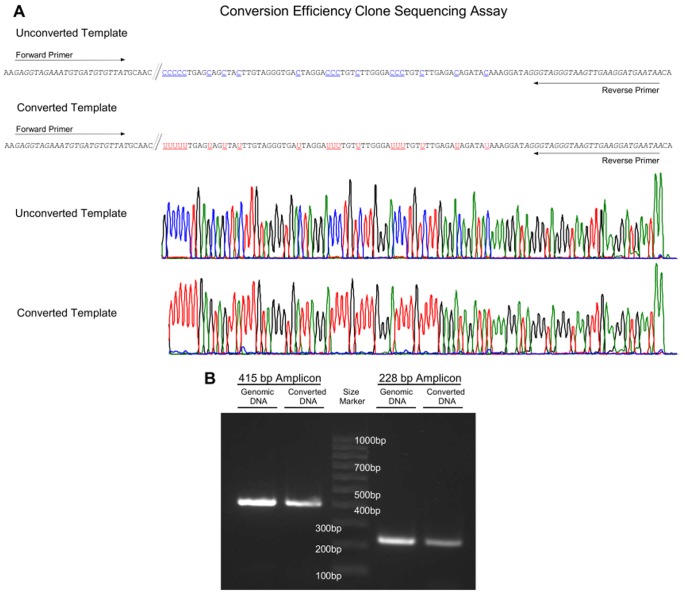
Principle of the CFP Clone Sequencing Assay. **A:** Basic principle of the CFP clone sequencing assay for quantifying the bisulfite conversion efficiency of the bisulfite kits. The two conversion-unspecific oligonucleotides bind to genomic sites which do not contain any cytosines and therefore amplify converted, partly converted, and unconverted DNA.

**Figure 8:**
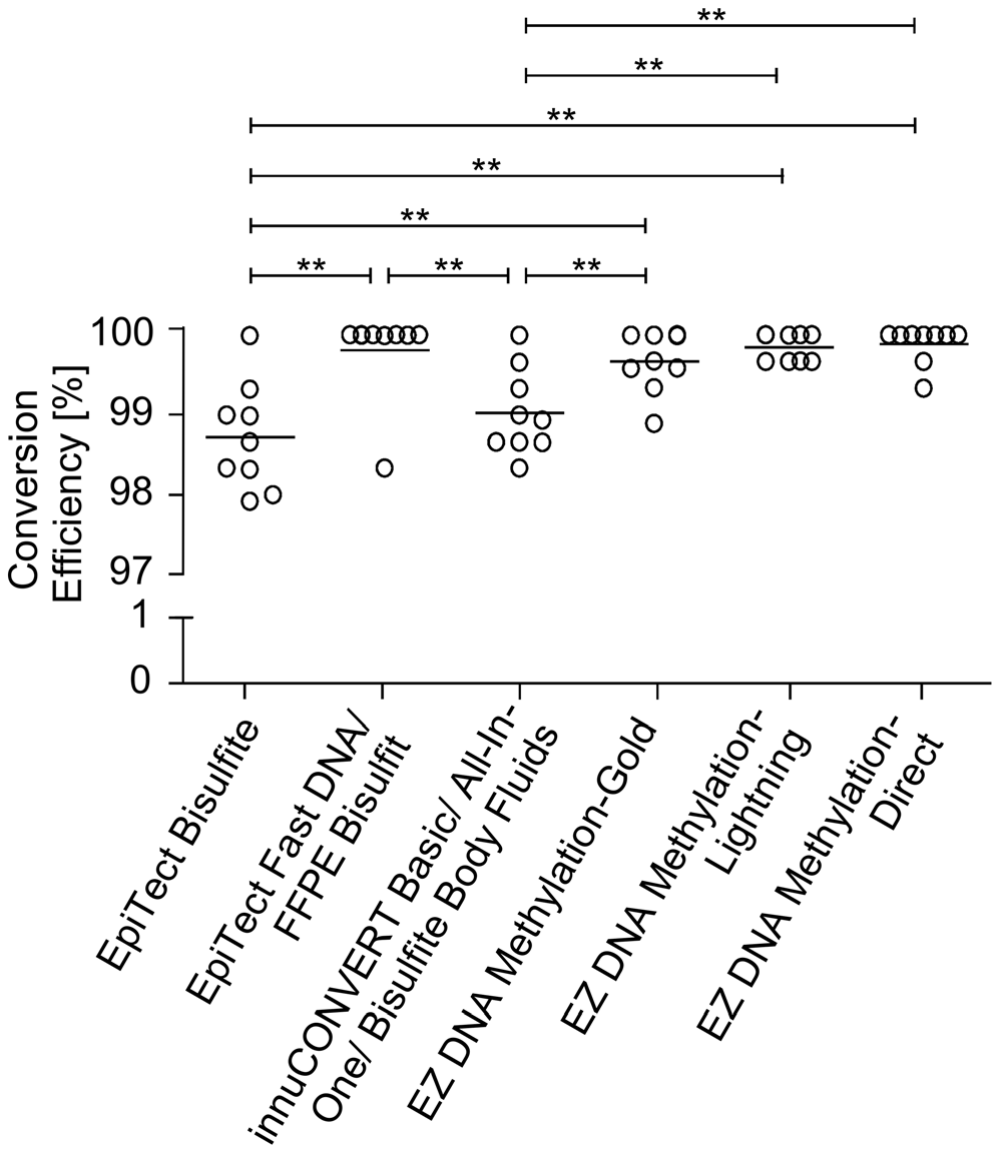
Conversion Efficiency of Different Bisulfite Conversion Kits. DNA was bisulfite converted using nine different commercially available kits. Each kit was processed in nine independent bisulfite reactions using 2 μg HMW input DNA, each. The specific conversion of cytosines to uracils at two different genetic loci were analyzed by means of the CFP clone sequencing assay.

The conversion efficiency of each kit was tested by means of clone sequencing of a PCR product generated using primers targeting cytosine-free priming (CFP) sites (**Figure 7** **A**). The CFP sites do not comprise any cytosines and therefore are not altered during the bisulfite conversion. Hence, unconverted and converted DNA is amplified with the same efficiency. Accordingly, a bias toward the amplification of completely converted DNA and therefore an overestimation of the conversion efficiency is avoided. The conversion-unspecific amplification of two primer pairs, encoding a 415 bp- and a 228 bp-fragment located in different regions of chromosome 2, were shown to amplify genomic as well as bisulfite converted DNA (**Figure 7** **B**). The CFP assay was used to quantify the specific conversion of cytosine to uracil. Altogether 421 clones comprising 18900 conversion sites were analyzed. Conversion rates above 98.7% were obtained with all kits (**Figure 8**). The EZ DNA Methylation-Direct Kit showed the highest conversion rate of 99.9%. In comparison, the EpiTect Bisulfite Kit showed the lowest conversion rate of 98.7%.

**Figure 9:**
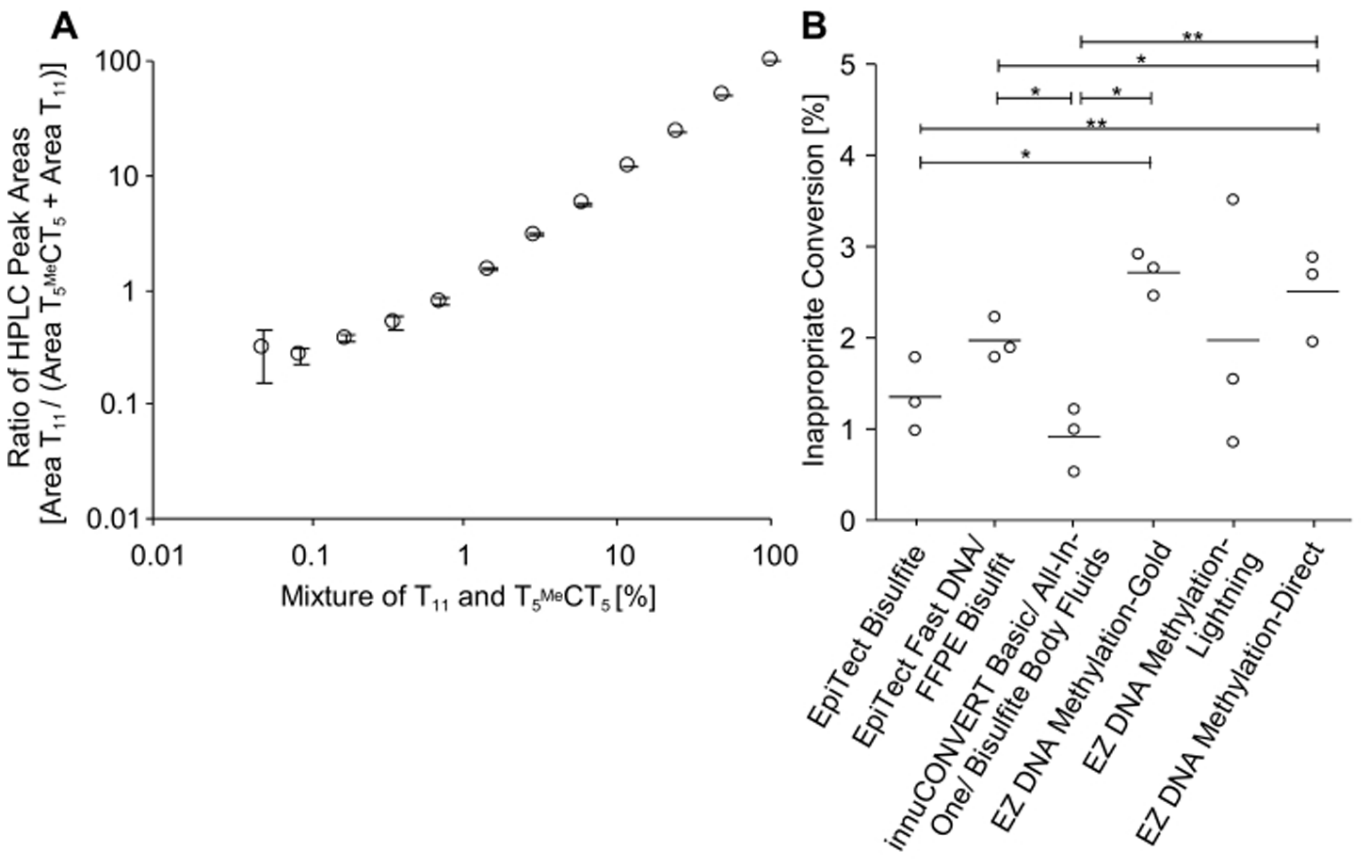
Inappropriate Conversion of Methylated Cytosines to Thymines. **A:** A series of dilutions of T_11_ in T_5_^Me^CT_5_ was analyzed in the reversed-phase HPLC to determine the detection and quantification limits of the HPLC at low concentrations of T_11_. The percentage of T_11_ in the mixture of T_11_ and T_5_^Me^CT_5_ was determined by calculating ratio of the respective peak areas (area T_11_/(area T_11_+ area T_5_^Me^CT_5_)) **B:** The inappropriate conversion of methyl-cytosine to thymine was determined for nine different commercially available bisulfite conversion kits. Each bisulfite reaction was performed in triplicate.

Whereas the conversion efficiency of cytosines to uracils should optimally be 100% the inappropriate conversion of methylated cytosines to thymines should be as low as possible. This inappropriate conversion was studied using an 11-mere DNA oligonucleotide containing a centred methylated cytosine (T_5_^Me^CT_5_). The inappropriate conversion of this oligonucleotide resulting in T_11_ was quantified by HPLC. As shown in **Figure 9** **A**, the analytical performance evaluation of the HPLC analysis revealed a limit of quantification below 1% T_11_ in the mixture of T_11_ and T_5_^Me^CT_5_. The kits led to an inappropriate conversion between 0.9% (innuCONVERT kit family) and 2.7% (EZ DNA Methylation-Gold Kit) (**Figure 9** **B**). A significant correlation between conversion efficiency and inappropriate conversion (Pearson’s correlation coefficient 0.83, p = 0.04) of the used protocol was found. This is expected since a bisulfite treatment under harsh conditions leads to a more complete conversion on the one hand but to an increased inappropriate conversion on the other hand.

### Direct Conversion Performance on Different Sample Materials

Three kits were compared (EpiTect Fast FFPE Bisulfite Kit, innuCONVERT Bisulfite All-In-One Kit, and EZ DNA Methylation-Direct Kit) that are applicable for the direct input (without prior extraction) of sample material, such as fresh tissue, FFPE tissue or cultured cells. The kits were tested and compared for these applications.

**Figure 10:**
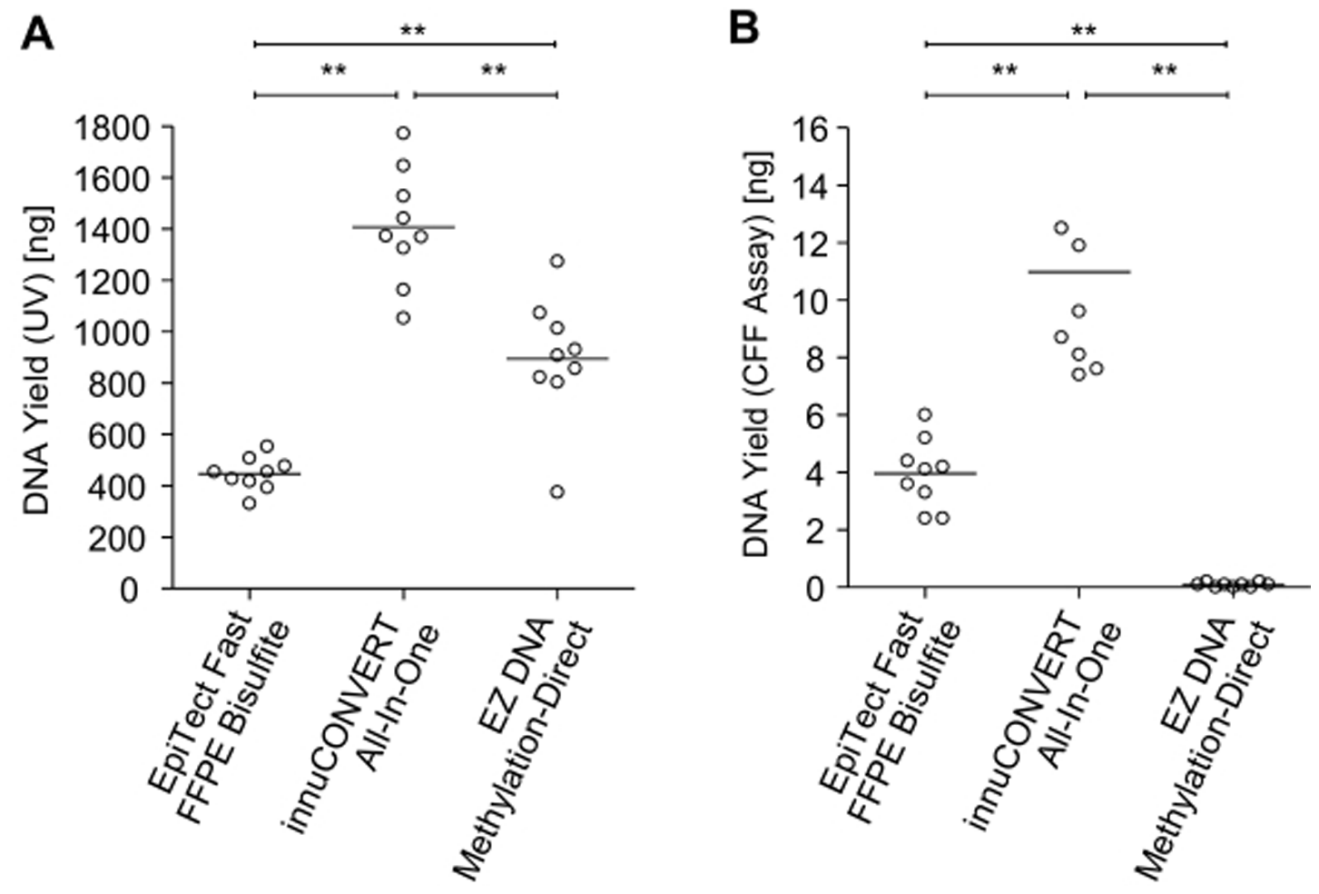
Direct Bisulfite Conversion of FFPE Tissue Sections without Prior Extraction. Total yield of bisulfite-converted DNA derived from the direct processing of 10 μm FFPE placental tissue sections using three bisulfite conversion kits. Each kit was tested in 9 replicates. Shown are mean values (± standard deviation) of triplicate CFF qPCR measurements (**B**) and results from a single UV measurement (**A**), respectively.

For the direct application of FFPE tissue sections each kit was tested with an input of one 10 μm section of FFPE placental tissue. The samples were analyzed as described above by UV and CFF qPCR assay. DNA prepared using the innuCONVERT Bisulfite All-In-One Kit resulted in a significantly higher yield compared to the other direct kits (**Figure 10**). DNA prepared from FFPE tissues by means of EpiTect Fast FFPE Bisulfite Kit and innuCONVERT Bisulfite All-In-One Kit showed high performance in downstream PCR application while bisulfite DNA generated using the EZ DNA Methylation-Direct Kit showed a poor performance during PCR amplification.

**Figure 11:**
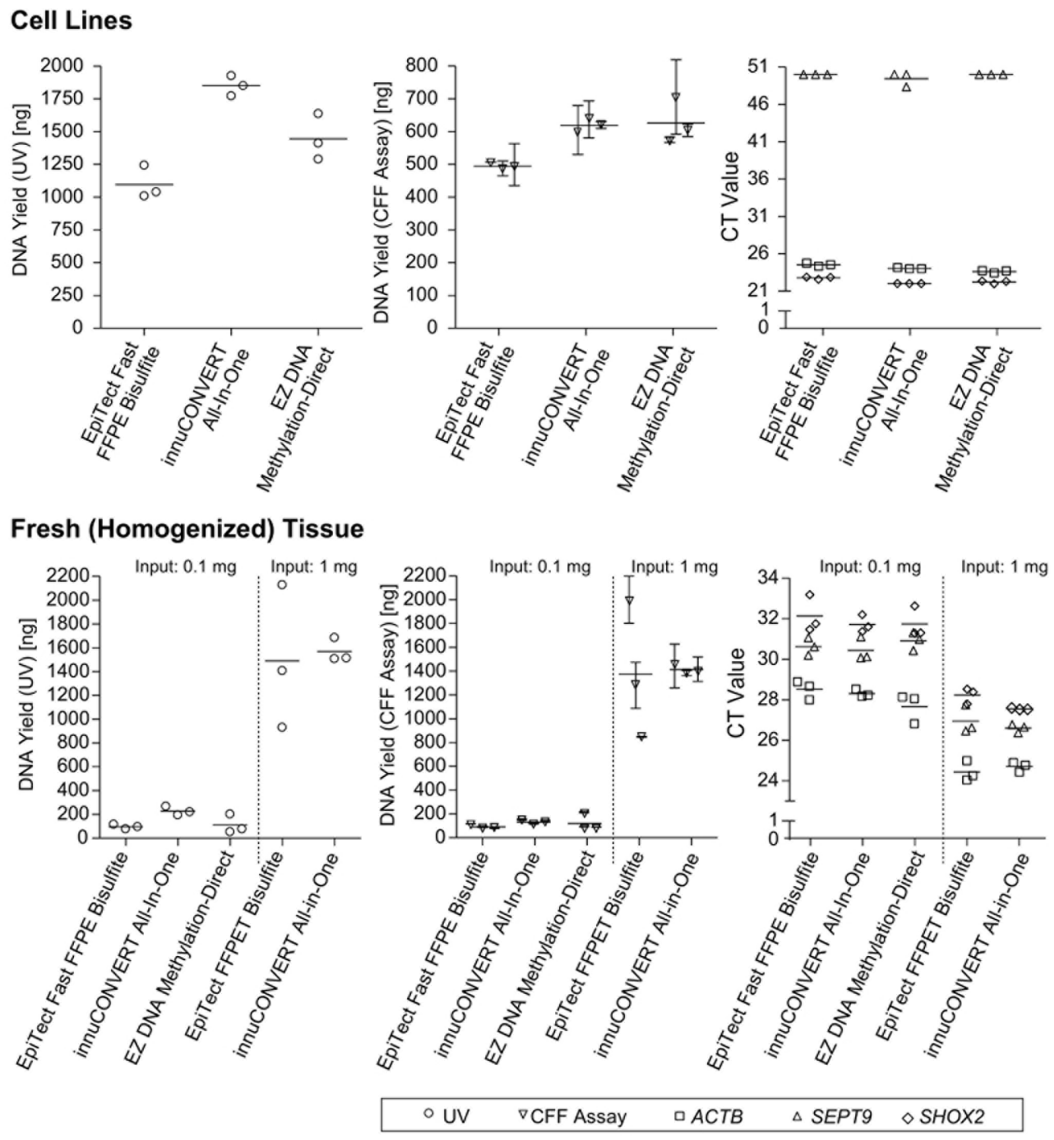
Direct Input of Samples Without Prior Extraction. Yield of bisulfite-converted DNA obtained from fresh (homogenized) placental tissue (0.1 mg and 1 mg input, **lower panel**) and cultured cells (10^5^ cells, **upper panel**) as determined by UV measurement (**left panel**), CFF qPCR (**middle panel**) and *ACTB/SEPT9*/*SHOX2* triplex qPCR assay (**right panel**), respectively. Three different commercially available kits were tested in triplicate. The EZ DNA Methylation-Direct kit only allowed for the input of max. 0.1 mg tissue according to the manual. Shown are mean values (± standard deviation) of triplicate qPCR measurements and single UV determinations, respectively.

Furthermore, the colorectal adenocarcinoma cell line DLD1 (American Type Culture Collection (ATCC), VA, USA) and fresh (homogenized) placental tissue were used to test the direct input of fresh tissue and cell line samples without prior extraction. The DNA yield was determined by UV spectrophotometry, CFF real-time PCR assay and *SHOX2/SEPT9/ACTB* real-time PCR assay. According to UV spectrophotometry the innuCONVERT Bisulfite All-In-One Kit showed the highest DNA yield both when applying cultured cells and fresh (homogenized) tissue (**Figure 11**). When applying cultured cells the EpiTect Bisulfite Kit showed the lowest DNA yield (**Figure 11**). However, according to the kit manual the EZ DNA Methylation-Direct Kit did not allow for the preparation of 1 mg tissue.

### High Volume Samples (Plasma, Serum, Urine, Pleural Effusion, Ascites)

**Figure 12:**
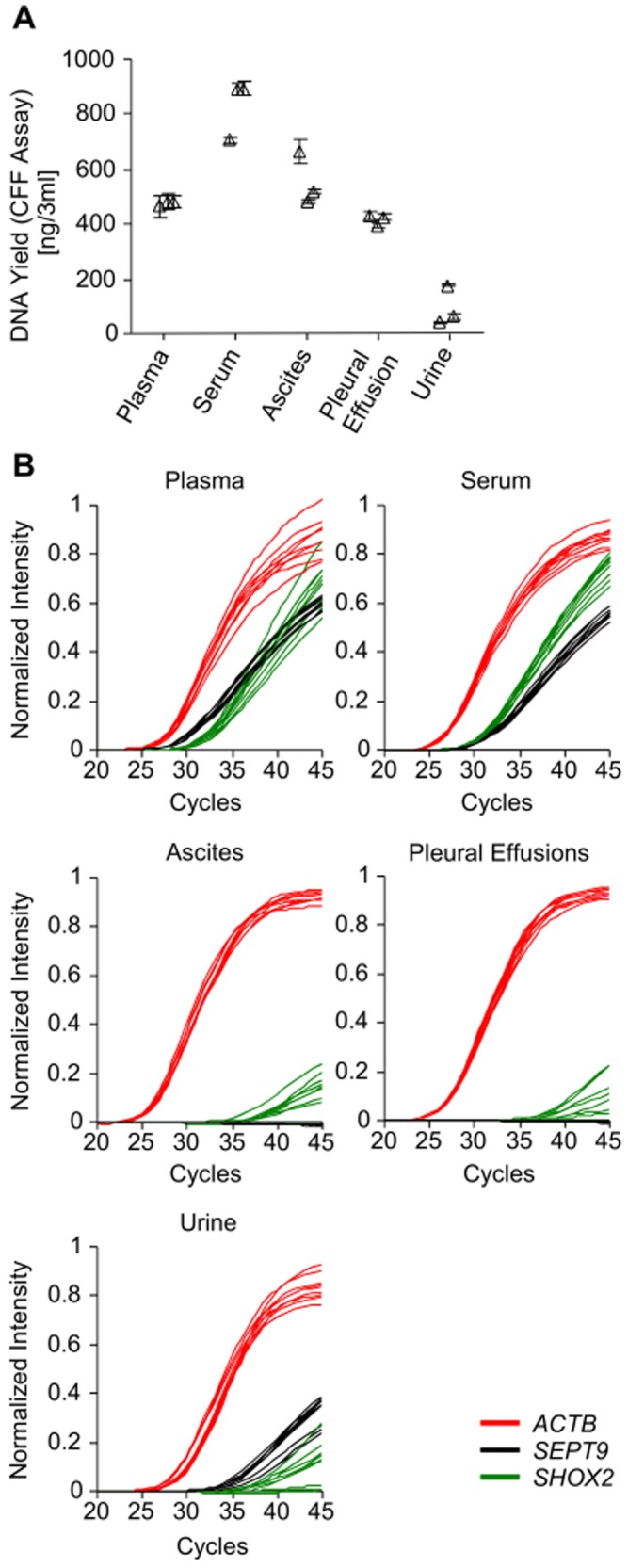
Bisulfite DNA from High Volume Body Fluids. Yield of bisulfite-converted cell-free DNA from various body fluids (urine, blood plasma and serum, ascites, and pleural effusions) as prepared using the innuCONVERT Bisulfite Body Fluids Kit. Bisulfite conversion of each sample type was done in three independent reactions. PCR measurements of each bisulfite reaction were carried in triplicate. **A**: Total DNA yield as quantified by the CFF qPCR assay. Shown are mean (± standard deviation) values of triplicate measurements. **B**: Amplification plots of the methylation-specific *ACTB*/*SHOX2*/*SEPT9* qPCR.

The innuCONVERT Bisulfite Body Fluids Kit was applied to 3 ml of plasma, serum, urine, and the supernatants of pleural effusion, and ascites samples. The DNA yield was quantified by the CFF and the *SHOX2*/*SEPT9*/*ACTB* quantitative real-time PCR assay. The innuCONVERT Bisulfite Body Fluids Kit allowed for the extraction of amplifiable DNA from all tested fluidic samples (**Figure 12**). Highest DNA yields were observed in serum samples (297 ng/ml). Plasma and the supernatants of ascites and pleural effusions showed DNA yields of 160 ng/ml, 173 ng/ml, and 142 ng/ml, respectively. In urine the DNA yield was lowest (21 ng/ml).

## Discussion

Several kits for bisulfite conversion of DNA are commercially available each showing advantages and disadvantages. The choice of a suitable kit for a specific application is not trivial and should be based on the specific performance requirements with regard to the respective sample material and biological/clinical question. While hands-on-time and time to result are usually rather an issue for routine molecular diagnostics, overall high yield of DNA suitable for downstream molecular analyses is mandatory in the fields of research and diagnostics. Furthermore, the occurrence of bisulfite conversion errors [27], [28] is an important parameter and should be considered carefully.

All nine kits studied in this series of experiments showed significantly different but comparable results and high performance when applying high molecular weight (HMW) DNA. Differences within the performance where more pronounced when applying degraded DNA from FFPE tissues. The loss of fragmented DNA during the purification procedure is most likely due to binding conditions which are optimized with regard to HMW DNA.

The bisulfite conversion is a chemical reaction under harsh chemical conditions (low pH, high temperature and elongated incubation times) causing significant DNA degradation [29], [30], [31]. Hayatsu summarized the principle of the bisulfite reaction [32]. In brief, bisulfite reacts with cytosines in a single stranded DNA molecule through the formation of a 5,6-dihydrocytosine-6-sulfonate intermediate preferably at acidic pH around pH 5. After HSO_3_^–^induced hydrolytic deamination of the intermediate [32], [33], bisulfite is eliminated at alkaline pH [32]. The optimal reaction conditions result from a balanced control of all desired (conversion of cytosines) and undesired reactions (DNA degradation and inappropriate conversion). In summary, high bisulfite concentrations and high temperature at prolonged incubations times will lead to a complete conversion of all cytosines to uracils on the one hand but will cause DNA degradation and inappropriate conversion of methylated cytosines to thymines on the other hand [27]. DNA degradation is mainly caused by depurination and depyrimidation leading to abasic sites [29], [31], followed by DNA strand breaks due to N-glycoside bond cleavage. Furthermore, prolonged incubation times lead to an increase of the time-to-results, therefore hampering the application of respective protocols for routine diagnostic purposes where rapid analyses are required to trigger clinical decisions. The methods for measuring bisulfite conversion reaction rates as described herein might be used in the future to identify the optimal reaction conditions allowing for sufficient conversion of cytosines but leading to only limited inappropriate conversion of methylated cytosines. Therefore, protocols and kits could be further improved.

The respective bisulfite chemistry is mainly responsible for fragmentation. Kits based on long incubation times lead to a higher fragmentation of DNA. These kits might show loss in performance when the analysis of larger PCR fragments is desired. Conventional bisulfite conversion protocols require hours of exposure to low-molarity, low-temperature bisulfite. Alternatively, high-molarity, high-temperature protocols have been developed by Hayatsu and co-workers [22], [23], [24], [32]. Genereux *et al.* [27] provide a comprehensive analysis and discussion of the two types of bisulfite-conversion errors: failure of conversion of unmethylated cytosines to uracils and inappropriate conversion of methylated cytosines to thymines. These errors will lead to false positive and false negative methylation signals, respectively, in downstream analyses. According to Genereux *et al.* [27] these high-molarity and high-temperature conditions are preferable because they yield greater homogeneity of bisulfite conversion, and thus yield more reliable data.

Genereux *et al.* [27] suggest that different durations of bisulfite treatment will yield data appropriate to address different experimental questions. Accordingly, the user is able to adjust the conditions in a way to either increase conversion of unmethylated cytosines or to reduce the inappropriate conversion of methylated cytosines. Another critical parameter is the stability of bisulfite solutions. In particular ammonium bisulfite is a strong reduction agent and therefore suffers from oxidation during prolonged exposure to oxygen. However, Genereux *et al.* [27] found only slightly decreased performance of bisulfite solutions which have been stored for 22 months after first opening. The innuCONVERT Bisulfite kits, the EpiTect Fast FFPE and Fast DNA Bisulfite kits and the EZ DNA Methylation-Lightning Kit are based on bisulfite solutions as core reagent and stability might represent a major shortcoming of these products. However, EpiTect Fast FFPE and Fast DNA Bisulfite kits and innuCONVERT Bisulfite kits contain several aliquots of bisulfite solutions and the user is recommended to discard the bisulfite solution after first use in order to avoid a performance loss.

All kits yielded highly pure DNA suitable for PCR analyses without inhibiting the PCR. DNA conversion efficiencies determined in this study ranged from 98.7% (EpiTect Bisulfite Kit) to 99.9% (EZ DNA Methylation-Direct Kit), which is a sufficiently high conversion for basically all biological and clinical applications. At the same time, the kits resulted in only moderate inappropriate conversion between 0.9% (innuCONVERT Bisulfite kits) and 2.7% (EZ DNA Methylation-Direct Kit). These figures are in concordance with a previous study, which reported inappropriate conversion rates between 0.09% and 6.1% [27]. It should to be considered that the conversion efficiency of cytosines depends on the sequence context [27]. Accordingly, slightly different results can be expected when using a different test system, i.e. different oligonucleotides. However, even though the performance differences between the kits regarding specific and inappropriate conversion are statistically significant, these differences seem not to be high enough to impair the downstream analysis of bisulfite DNA prepared with either kit. However, appropriate adaptions to the single protocols (higher incubation temperature, higher bisulfite concentration, prolonged incubation time) will allow increasing the conversion efficiency at the expense of higher inappropriate conversion.

All DNA samples were stable for at least 4 weeks when stored at −80°C to 4°C. A lack of stability and a degradation of bisulfite converted DNA due to mid-term storage (up to one month) at temperatures between −80 and 4°C was not found in this study. This is in concordance with previous findings where bisulfite-converted DNA gave similar results compared to freshly prepared bisulfite-converted DNA after storage of more then 2 years at −20°C [7]. Differences in storability of DNA at a higher temperature (37°C) are most likely due to the buffer conditions of the elution buffer. Accordingly, long-term storage of bisulfite-converted DNA might be possible when choosing appropriate storage buffer conditions.

Handling and user friendliness of the different kits differ significantly. Time-to-result ranged from 131 min (innuCONVERT kits) to 402 min (EpiTect Bisulfite Kit). Hands-on-time was between 66 min (EZ DNA Methylation-Lightning Kit) and 104 min (EpiTect Fast FFPE Bisulfite Kit and EpiTect Fast DNA Bisulfite Kit). Accordingly, the availability of labor and the necessity to obtain quick results might influence the choice of a suitable kit. The EpiTect and the EZ DNA Methylation kit product families require a thermal cycler which is not necessarily present in the pre-PCR area of each laboratory. Therefore, the utility of these kits is limited.

The most significant performance differences between the kits were found when applying sections from FFPE tissues directly without prior DNA extraction. The EZ DNA Methylation-Direct Kit failed to yield PCR-amplifiable bisulfite converted DNA from FFPE tissue sections while the other kits (EpiTect Fast FFPE Bisulfite Kit and innuCONVERT Bisulfite All-In-One Kit) yielded sufficient amount of PCR-amplifiable bisulfite DNA. Since the three direct kits did not show these high performance differences when applying extracted DNA (HMW and FFPET DNA), this effect is most likely due to the lysis protocol of the kits. The appropriate lysis is essential with regard to performance in downstream molecular applications [33]. Insufficient lysis will not only lead to low DNA yields but will also cause bisulfite artefacts, i.e. false positive methylation sites [28]. Increasing the duration of proteinase K treatment up to 48 hours and supplementing the reaction with additional proteinase K during the lysis will help to increase the yield of highly integer DNA [20], [34], [35], [36]. Furthermore, the introduction of an incubation step at high temperature after proteinase K treatment might help to remove remaining crosslinks based on the antigen retrieval principal and therefore lead to higher quality DNA [37]. Besides the highest yield of PCR-amplifiable bisulfite DNA the innuCONVERT Bisulfite All-In-One Kit is the only tested kit for the preparation of bisulfite DNA from FFPE tissues which does not require a preceding deparaffination of the tissue. Accordingly, this kit is of particular utility for critical sample materials where a deparaffination and re-hydration by means of xylene and ethanol series might lead to a loss of tissue.

The innuCONVERT Bisulfite All-In-One Kit showed the highest versatility regarding different input sample materials (DNA, FFPE tissues, cell lines, fresh and frozen tissues, cellular fractions of bronchial aspirates, pleural effusions, ascites, and urine sediment). There is an emerging need for protocols and kits that allow for the preparation of bisulfite DNA from low abundance biomarkers in high volume body fluidic samples of clinical relevance, i.e. urine, blood plasma and serum, ascites and pleural effusions. Several studies describe the sensitive analysis of DNA methylation biomarkers in plasma or serum as promising tests for early detection of various tumors [10], [11]. The low abundance of tumor DNA in blood, especially in blood from patients with early stage tumors where screening is particularly valuable, necessitates the usage of high volumes of plasma or serum in order to increase the likelihood of presence of methylated tumor DNA and therefore reach sufficient sensitivity of the test. To date the only commercially available kit enabling the preparation of bisulfite DNA from high volume (up to 3 ml) body fluid is the innuCONVERT Bisulfite Body Fluids Kit. Accordingly, performance comparison of different available kits is not possible. However, the yield of DNA in plasma and serum are in line with concentrations described in the literature with serum showing higher yields as compared to plasma [38], [39], [40].
